# Feasibility of a Modified Bronchoscopic Transparenchymal Nodule Access Technique (‘Essen Tunnel’) for Improving the Diagnosis of Intraparenchymal Pulmonary Lesions

**DOI:** 10.1111/resp.70053

**Published:** 2025-05-21

**Authors:** Erik Büscher, Faustina Funke, Jane Winantea, Hanna Zellerhoff, Johannes Wienker, Marcel Opitz, Christian Taube, Kaid Darwiche

**Affiliations:** ^1^ Department of Pulmonary Medicine, Division of Interventional Pneumology University Hospital Essen ‐ Ruhrlandklinik Essen Germany; ^2^ Institute of Diagnostic and Interventional Radiology and Neuroradiology University Hospital Essen Essen Germany; ^3^ Department of Pulmonary Medicine University Hospital Essen ‐ Ruhrlandklinik Essen Germany

**Keywords:** bronchoscopy, diagnostic tool, lung cancer, lung cancer screening, solitary pulmonary nodule

## Abstract

**Background and Objective:**

Diagnosing intraparenchymal pulmonary lesions lacking a bronchus sign remains challenging. Bronchoscopic transparenchymal nodule access (BTPNA) for reaching such lesions has seen limited clinical adoption due to insufficient evidence and practical challenges. This study evaluates the feasibility and diagnostic yield of a modified BTPNA (mBTPNA) technique—referred to as the ‘Essen tunnel’—which eliminates the need for a guide sheath.

**Methods:**

A retrospective analysis was conducted on patients undergoing virtual bronchoscopic navigation (VBN) incorporating the mBTPNA technique at our centre, from December 2019 to March 2024. The ‘Essen tunnel’ was created by a needle under virtual navigation guidance, enabling direct insertion of an ultrathin bronchoscope (UTB) or radial endobronchial ultrasound (R‐EBUS) probe to biopsy intraparechymal lesions.

**Results:**

Among 266 lesions targeted via VBN, 37 (14%) intraparenchymal lesions (mean target length: 12.7 ± 4.1 mm) were accessed using mBTPNA. The tunnel was successfully created in 97.3% of cases with UTB intubation in 51.4%. R‐EBUS was inserted into the tunnel in 83.8% of cases. Semicircular to circular patterns (SCP) were detected in 19.4% before and 61.3% after tunnel creation (*p* < 0.01). SCP presence on R‐EBUS following mBTPNA was associated with a diagnostic accuracy of 66.7%, comparable to that observed in non‐tunnel lesions exhibiting a bronchus sign (72.9%, *p* = 0.58). No severe complications were observed.

**Conclusion:**

The mBTPNA technique is a feasible and safe method that enhances endosonographic lesion detection and achieves promising diagnostic accuracy for challenging intraparenchymal lesions.

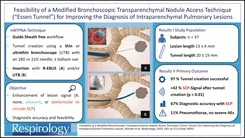

## Graphical abstract

The modified bronchoscopic transparenchymal nodule access technique, referred to as the ‘Essen Tunnel’, has demonstrated feasibility and significantly enhances endosonographic lesion detection, thereby improving diagnostic accuracy for challenging intraparenchymal lesions. Utilising an ultrathin bronchoscope for tunnel creation and subsequent intubation further extends lesion accessibility.

## 1 Introduction

The implementation of lung cancer screening is set to markedly enhance the detection of indeterminate nodules, thereby posing a significant challenge for clinicians. Transthoracic approaches are commonly employed for the diagnosis of peripheral nodules [1], but these methods have inherent limitations such as a high risk of pneumothorax [1, 2]. Another invasive approach is surgical resection, which requires a certain degree of physical fitness to undergo this procedure [3]. Endoscopic approaches, on the other hand, are less invasive and offer the advantage of a single procedure that can target peripheral lung lesions, perform mediastinal staging with endobronchial ultrasound, or even place fiducial markers [4].

In recent decades, virtual navigation bronchoscopy (VBN) has proven to advance the diagnosis of peripheral lung lesions and enhance the visualisation of lesions during biopsy [4, 5, 6, 7, 8, 9, 10]. However, VBN has a critical limitation: its diagnostic accuracy is reduced in scenarios where there is no bronchus sign to indicate direct access to the nodule via the airway. One promising technique to address this limitation is bronchoscopic transparenchymal nodule access (BTPNA), which aims to establish an artificial airway pathway through the lung parenchyma. This approach expands the range of available instruments and enhances access to the lesion, thereby improving diagnostic capabilities. Initially validated in animal models [11, 12], BTPNA was later described by Herth et al. in a first‐in‐human trial [13]. Since then, the technique has been adopted by other institutions [14, 15] and the idea of BTPNA has been integrated with alternative navigational technologies such as electromagnetic guidance, also in combination with cone beam CT [4, 16]. The traditional BTPNA procedure uses VBN and fused fluoroscopy to guide the biopsy through bronchial penetration at a central, endoscopically visible entry point. After dilation of the entry hole with a balloon, an atraumatic guide sheath (GS) is advanced to establish a pathway to the lesion. Once the nodule is reached under fluoroscopic guidance, the sheath's stylet is withdrawn to allow instrument insertion [13]. However, the evidence supporting BTPNA remains limited and its restricted practicality has prevented clinical adoption. Dependence on a GS and manufacturer‐provided access kits restrain bronchoscope size, confining its use to centrally located entry points. This poses significant challenges, particularly for left upper lobe lesions, where anatomical structures like the pulmonary artery and aorta complicate access [17]. Moreover, the GS's limited flexibility, manoeuvrability, and angulation significantly reduce its clinical utility, while the high material costs present an additional barrier to its integration into clinical practice.

We present our centre‐specific modified BTPNA (mBTPNA) approach, featuring modifications to address and potentially overcome the limitations of the traditional technique. In contrast, mBTPNA eliminates the need for a GS, allowing the direct introduction of instruments. Additionally, it facilitates the insertion of a radial endobronchial ultrasound (R‐EBUS) probe or an ultrathin bronchoscope (UTB) into the transparenchymal tunnel. This study aims to evaluate the feasibility and potential for improving the endosonographic lesion detection and diagnostic accuracy of intraparenchymal lesions using the modified BTPNA approach, termed the ‘Essen Tunnel’.

## 2 Methods

### 2.1 Procedure

Subjects undergoing VBN supplemented by fused fluoroscopy using the Archimedes system (Broncus Medical Inc., United States) from December 2019 to March 2024 were retrospectively analysed, with a particular focus on the mBTPNA technique, referred to as the ‘Essen Tunnel’. A detailed description of the planning and standard procedure of VBN can be found in the literature [14, 18]. The study was conducted in accordance with the guidelines of the Declaration of Helsinki and received approval from the institutional review board (24‐11,881‐BO).

**FIGURE 1:**
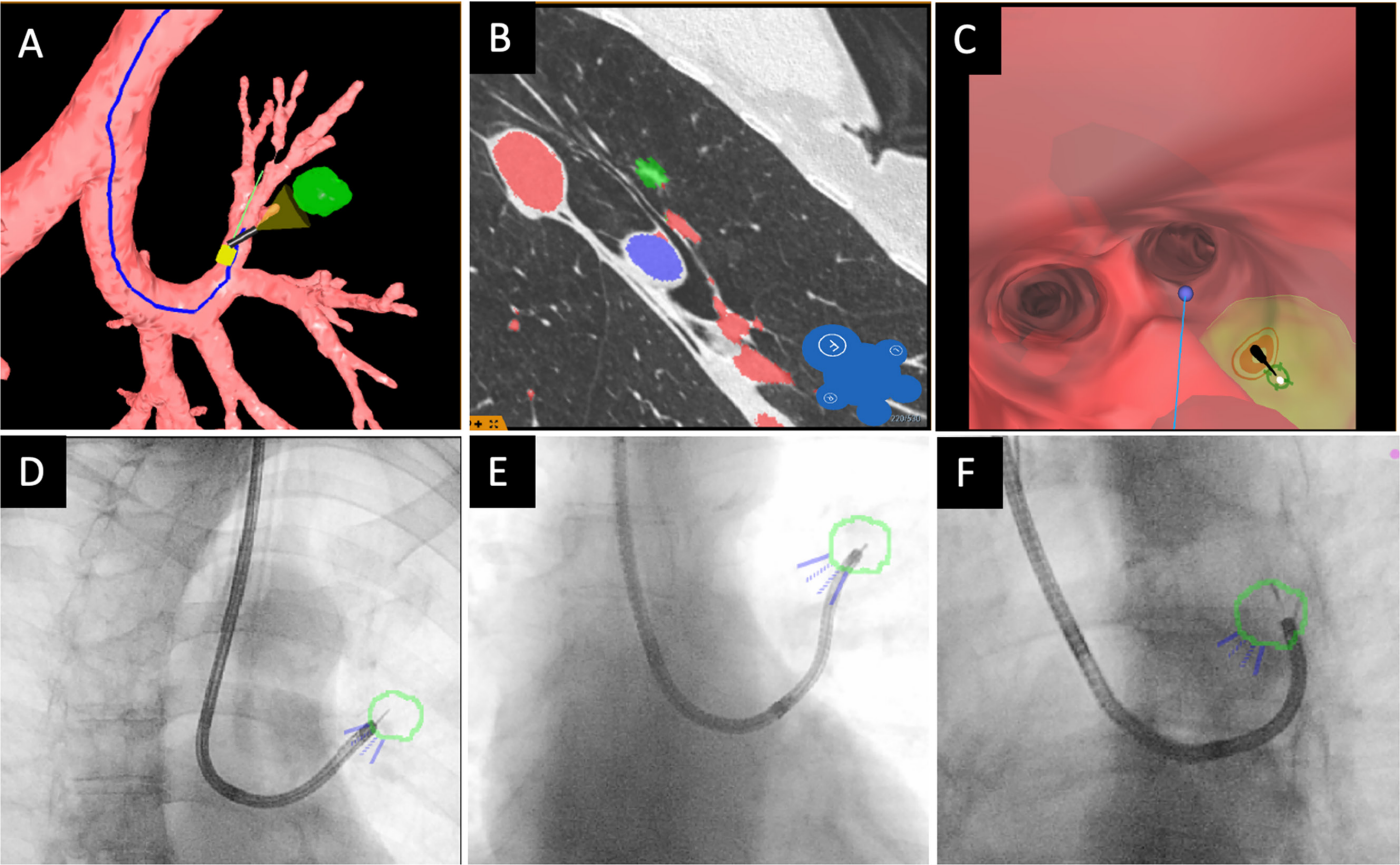
Virtual navigation planning (A–C) and intraoperative real‐Time biopsy guidance with fused fluoroscopy (D–F). A: View of the virtual 3D reconstruction with the marked navigation path (blue line) leading to the target lesion in the left upper lobe (green area). B: Annotation of vascular structures enables the activation of the virtual Doppler function (C) within the endoscopic virtual navigation view. The target lesion is visible as a greenish area, with the red target marker centrally indicating the point of entry for tunnel creation. The reddish structures visible through the bronchial mucosa represent vascular structures. D–F: Fused fluoroscopy with the target lesion (green segmentation) derived from the pre‐procedural CT scan and VBN segmentation. Utilising a C‐arm, the biopsy tool is positioned within the virtual target in three different planes, indicating successful virtual target attainment.

**FIGURE 2:**
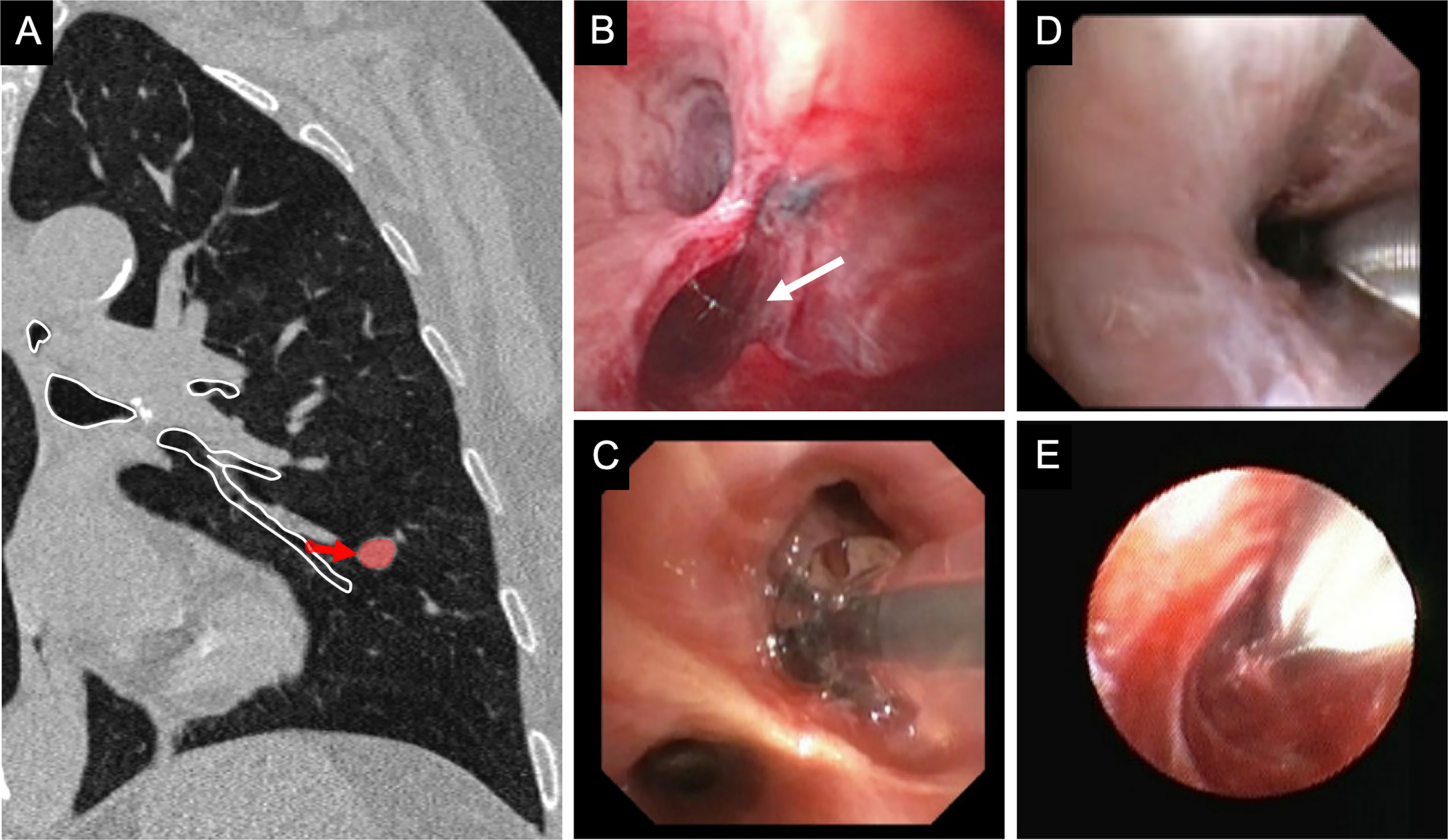
(A) Intraparenchamally located lesion (highlighted in orange) in the lingula, as seen in the coronal CT view. White lines indicate the airways, and the red arrow represents the transparenchymal route from the Point of Entry, measuring 11 mm. Endoscopic views: (B) Entrance of the Essen Tunnel, (C) balloon dilation, (D) insertion of the R‐EBUS probe into the tunnel and (E) perspective of an ultrathin bronchoscope on the target lesion within the Essen‐Tunnel prior to sampling.

Lesions with intraparenchymal localization, where the estimated point of entry (POE) was within a suitable central, segmental, or subsegmental bronchus and the tunnel path free of interfering vessels, were considered eligible for tunnel creation. During the planning phase, the Archimedes system was used to calculate the optimal navigation path (Figure 1A). By annotating the vascular structures (Figure 1B), the virtual Doppler function (Figure 1C) was employed to identify a vessel‐free tunnel path extending from the POE. During the procedure, the virtual view served as a navigation aid. Intraoperatively, R‐EBUS was utilised to confirm the absence of vessels along the tunnel path in real time. Endoscopic visualisation of the entrance to the airwall penetration site was a prerequisite. Choice of bronchoscope size was upon the lesion site and corresponding accessibility of the POE via either a thin or an ultrathin bronchoscope (BF‐P190 with 4.2 mm outer diameter or BF‐MP‐190 with 3 mm outer diameter, Olympus, Japan), while the narrower scope diameter is postulated to access up to two branches beyond the BF‐P190 accessible range. For lesions where centrally located vessels prevented direct transparenchymal tunnelling, the utilisation of an UTB to bypass these impeding structures through a more peripheral access was considered. Figure 2A illustrates a typical intraparenchymal lesion suitable for the Essen Tunnel with the POE located in the inferior lingula segmental bronchus.

**FIGURE 3:**
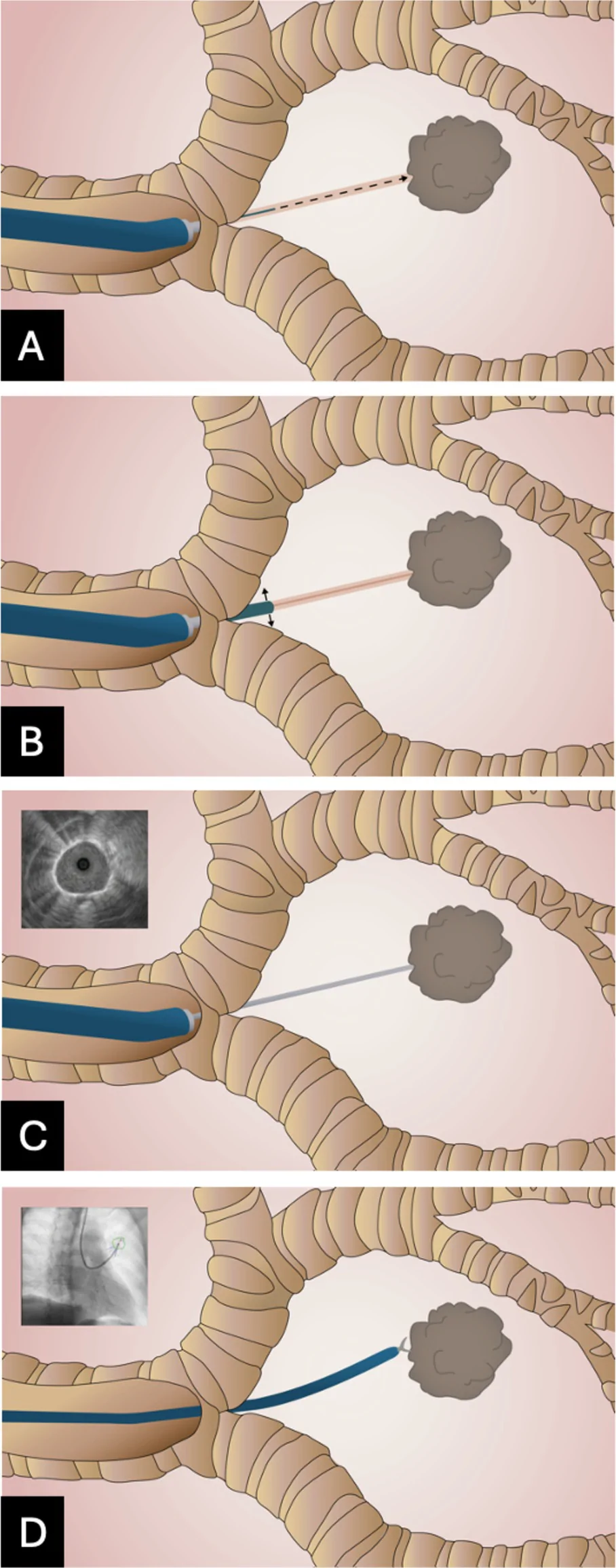
Schematic illustration of the procedural steps for creating an Essen Tunnel. (A) Perforation of the airway wall with a needle directed towards the target lesion; After this step, instruments, such as mini forceps or cryobiopsy, can be introduced through the tunnel. (B) Optional dilation of the tunnel entrance using a balloon, compatible only with the 4.2 mm endoscope. (C) Advancement of the R‐EBUS probe through the tunnel into the target area, demonstrating a circular signal as endosonographic confirmation of the tool in‐lesion. (D) Introduction of a 3 mm endoscope through the tunnel towards the target lesion for subsequent sampling, such as with mini forceps, guided by fused fluoroscopy.

The individual steps of the Essen Tunnel are shown in Figure 2B–E from an endoscopic perspective and schematically in Figure 3. Depending on bronchoscope size either a 21‐gauge (Periview needle, Olympus, Japan) or an 18‐gauge needle (Flexneedle, Broncus Medical Inc.) was used for perforation of the airway wall (Figure 3A). During the procedure, fused fluoroscopy was used for real‐time visualisation of the lesion and guidance of the instruments (Figure 1D–F). A tunnel sheath as previously described in the literature [13, 14] has not been utilised. Subsequent steps included one or a combination of the following methods:

- Atraumatic balloon dilatation (Single use Balloon Dialation Catheter 6 × 20 mm LeoMedical Co Ltd. China) when the entrance hole was inaccessible for further steps (Figures 2C and 3B).
- Insertion of a R‐EBUS Probe for endosonographic lesion confirmation (Figures 2D and 3C).
- The utilisation of biopsy instruments, including forceps, cryoprobe, TBNA, brush, or catheter, through the tunnel.
- The advancement of an UTB through the tunnel to minimise the distance to the lesion (Figures 2E and 3D).

After biopsy of the pulmonary lesion, subsequent mediastinal staging with linear endobronchial ultrasound was performed, as deemed necessary. After the procedure and subsequent transfer to the recovery room, all patients underwent a chest X‐ray to rule out pneumothorax and clinically relevant pulmonary haemorrhage.

### 2.2 Outcome

The primary aim of this study was to evaluate the feasibility and effectiveness of the modified BTPNA method (‘Essen Tunnel’) in enhancing endosonographic lesion detection rates and diagnostic yield. Specifically, the study sought to assess whether the mBTPNA procedure, including R‐EBUS insertion, could achieve a diagnostic yield for intraparenchymal lesions comparable to that of VBN in lesions where no transparenchymal tunnelling was performed, particularly for those exhibiting a bronchus sign. Secondary objectives focused on assessing the safety profile of the procedure.

**FIGURE 4:**
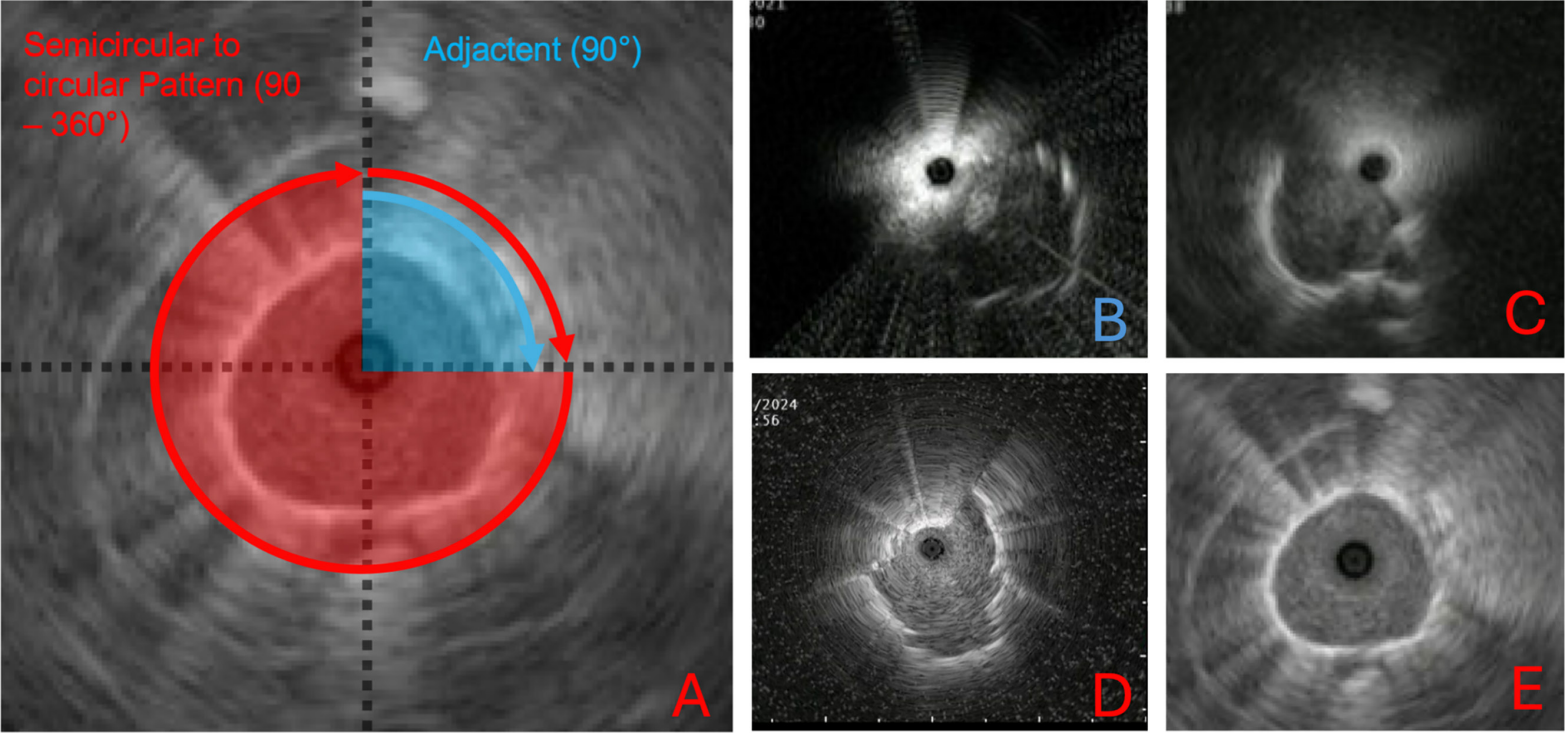
(A) The classification of endosonographic lesion patterns was based on the extent of the lesion signal. Semicircular, three‐quarter circular, and circular signals were collectively referred to as the semicircular to circular pattern (SCP). Eccentric patterns were categorised as follows: Adjacent (up to 90°, B), semicircular (90°–180°, C), and three‐quarter circular (180°–270°, D). A full 360° view was classified as circular (E).

Feasibility was assessed based on the number of successfully created tunnels, endoscopic intubations, and R‐EBUS insertions achieved within the mBTPNA‐tunnel. The classification of endosonographic images was based on the extent of the lesion signal. Patterns that deviated from a circular shape (> 270°) were classified as eccentric [19, 20]. The eccentric pattern was further subdivided into no signal, adjacent (up to 90°), semicircular (90°–180°) and three‐quarter circular (180°–270°), as illustrated in Figure 4. Semicircular, three‐quarter circular, and circular signals were collectively referred to as the semicircular to circular pattern (SCP). The endosonographic lesion detection rate was evaluated by comparing the R‐EBUS signals before and after mBTPNA, while an upgrade to the next signal pattern category was considered an improvement in the endosonographic signal.

Diagnostic accuracy was defined as the proportion of peripheral pulmonary lesions with biopsy results—whether positive or negative—that were confirmed by follow‐up [21]. Predefined criteria described by Avasarala et al. [22] were used to define a positive histological result. Histological results that did not meet the predefined criteria are classified as either non‐specific (e.g., inflammation, fibrosis) or non‐diagnostic (ND) (e.g., normal tissue, atypical cells). Nondiagnostic results were classified as false negatives without considering follow‐up data, and diagnostic accuracy was calculated as the proportion of correctly identified lesions (true positives and true negatives) relative to the total number of lesions, in accordance with the ‘intermediate’ approach described by Vachani et al. [23]. The minimum follow‐up requirement was at least 3 months or histologic confirmation through another minimally invasive sampling method or surgical resection. To evaluate the diagnostic accuracy, we performed analyses both including and excluding cases lost to follow‐up or not meeting the minimum follow‐up criteria [24]. Bleeding events during the procedures were classified according to the criteria established by the Nashville Working Group [25].

### 2.3 Statistical Analysis

Statistical analysis was performed using IBM SPSS Statistics version 29. Descriptive statistics were used, with categorical variables reported as frequencies (percentages) and quantitative variables reported as means (standard deviations) or medians (interquartile ranges). Missing data were excluded from the analysis and the number of missing data for each variable was reported. The Shapiro–Wilk test was used to assess the normal distribution of quantitative data. Parametric data were compared using the independent samples t‐test, while non‐parametric data were assessed using the Mann–Whitney *U* test. Categorical variables were compared using the chi‐squared test or Fisher's exact test, as appropriate, and the McNemar test was used for paired sample analysis. *p* values less than 0.05 were considered statistically significant.

## 3 Results

### 3.1 Study Participants and Lesion Characteristics

**FIGURE 5:**
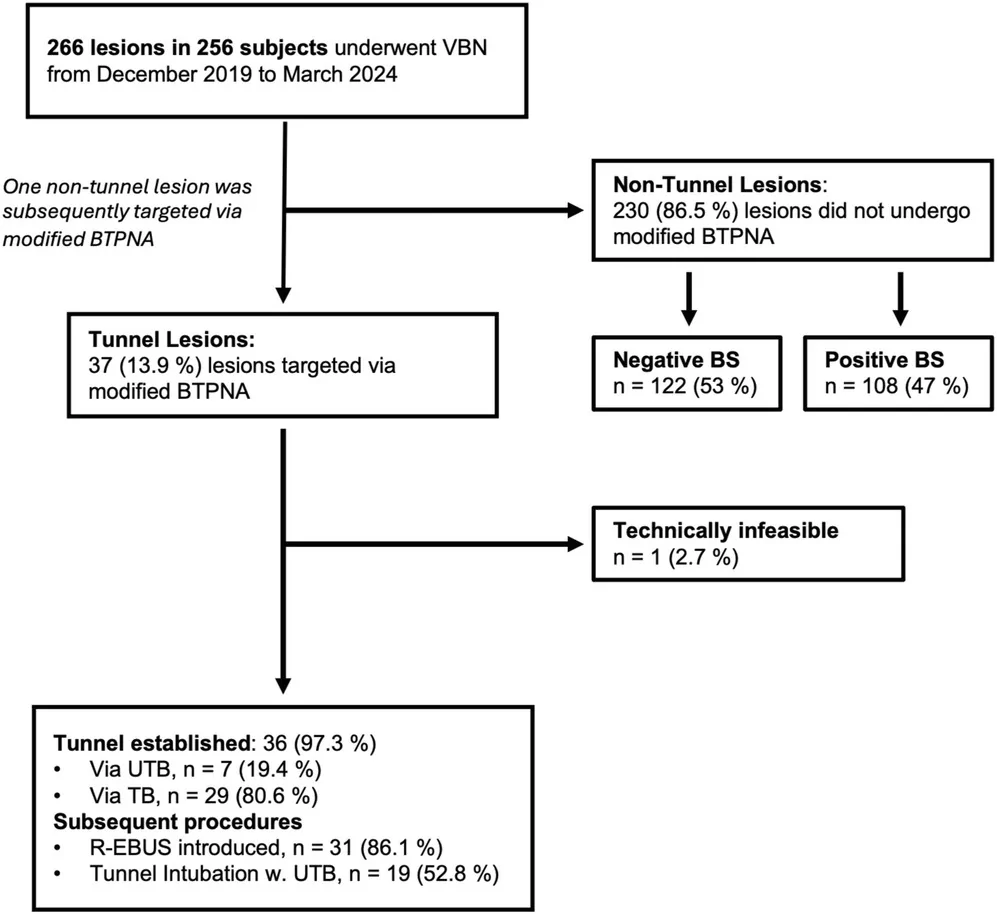
Flowchart illustrating study enrollment and procedures performed. BS, bronchus sign; BTPNA, bronchoscopic transparenchymal nodular access; R‐EBUS, radial endobronchial ultrasound; TB, thin bronchoscope; UTB, ultrathin bronchoscope; VBN, virtual bronchoscopic navigation; w., with.

Among the 266 lesions evaluated via VBN, 13.9% (*n* = 37) were targeted with the mBTPNA approach Essen Tunnel, while 86.5% (*n* = 230) were assessed without tunnel creation (Figure 5). In the mBTPNA technique group, 56.8% were female patients, with an average age of 68.4 years (± 10.6). Lesion analysis revealed a mean long‐axis diameter of 18.6 mm (± 6.5), with 59.5% of the lesions being smaller than 2 cm. The mean planned tunnel length was 20.3 ± 15.3 mm, while the target length at the puncture angle averaged 12.7 ± 4.1 mm, and the distance from the lesion to the pleura was 34.4 ± 22.3 mm. Detailed baseline characteristics and lesion parameters are presented in Table S1, with a comparison with those of non‐tunnel lesions.

### 3.2 Impact of R‐EBUS Guidance in Modified BTPNA on Diagnostic Accuracy

**FIGURE 6:**
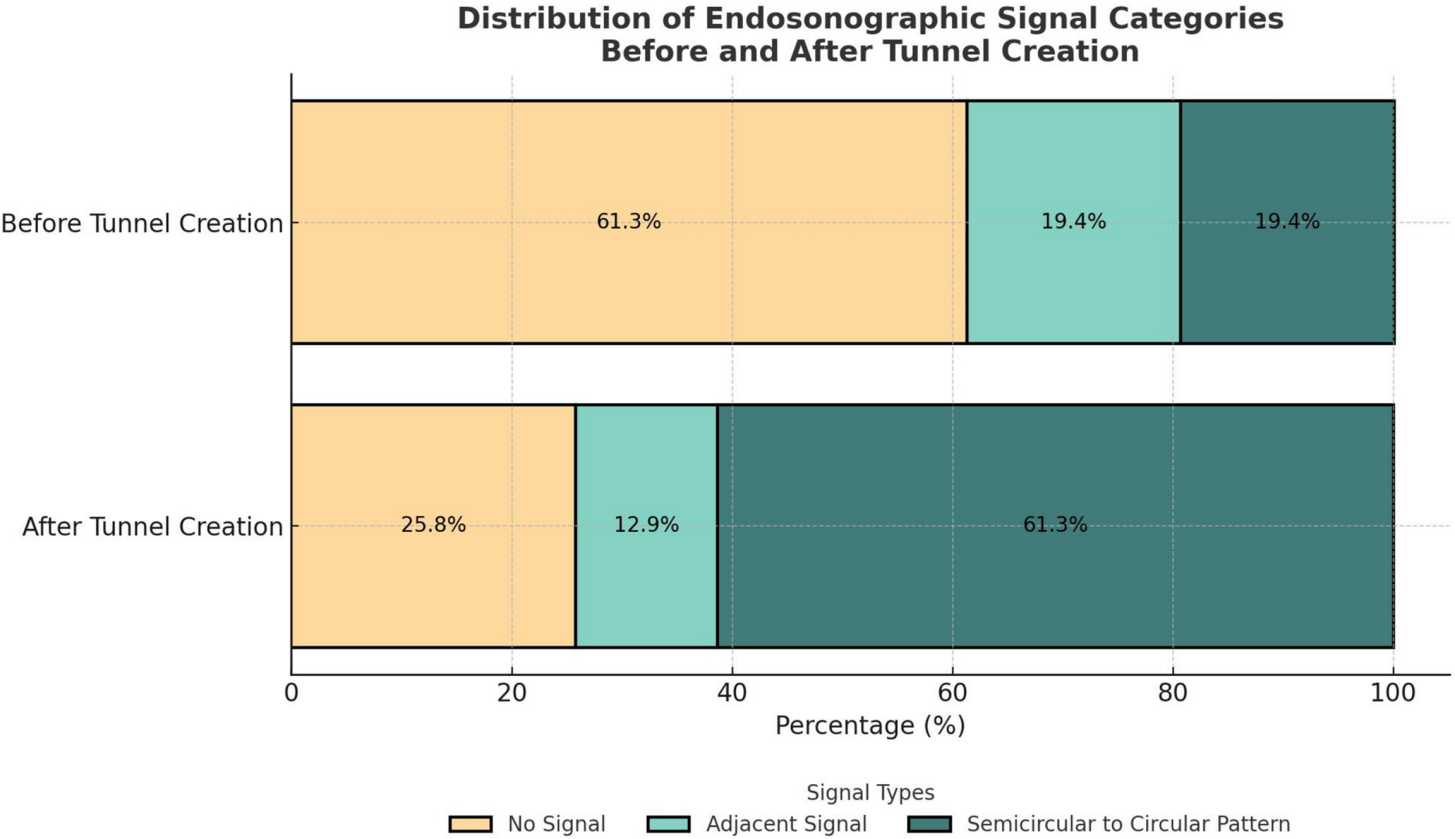
Distribution of endosonographic signal categories before and after mBTPNA for lesions with R‐EBUS Insertion via tunnel (*n* = 31); mBTPNA, modified BTPNA.

The R‐EBUS probe was successfully inserted into the tunnel in 83.8% of cases (31/37), improving endosonographic signal classification in 54.8% (17/31) of these procedures. Prior to tunnel creation, 61.3% of lesions with R‐EBUS tunnel insertion showed no signal, which significantly decreased to 25.8% after tunnel creation (*p* < 0.01). Furthermore, an SCP signal was observed in 19.4% of cases before tunnelling, increasing to 61.3% following mBTPNA tunnel creation (*p* < 0.01). The distribution of endosonographic signal categories is illustrated in Figure 6 and further detailed in Table S2.

For lesions without an R‐EBUS signal before tunnelling (*n* = 25), R‐EBUS insertion was performed in 84.0% (21/25), with signal detection achieved in 52.4% (11/21), of which 47.6% (10/21) exhibited an SCP. In lesions with pre‐existing signals (*n* = 12), R‐EBUS insertion was carried out in all cases, improving signal classification in 50.0% (6/12), with 66.7% (8/12) displaying an SCP via mBTPNA.

SCP presence significantly enhanced diagnostic accuracy, reaching 66.7% (12/18) versus 29.4% (5/17) for no/adjacent signals (*p* = 0.04). Circular patterns achieved the highest diagnostic yield at 83.3% (5/6). Lesions with bronchus signs targeted via VBN alone had a diagnostic yield of 72.9%, comparable to mBTPNA‐accessed lesions with SCP (*p* = 0.59). Cryobiopsy was performed in 12 cases (32.4%), with a diagnostic accuracy of 58.3% (7/12) for lesions biopsied using a cryoprobe, compared to 40% (10/25) for those biopsied without it. However, this difference did not reach statistical significance (*p* = 0.48). For lesions with benign or non‐specific histologic results (*n* = 169), the median follow‐up duration was 3.0 (1.0–8.0) months. Detailed diagnostic outcomes are summarised in Table 1 and Table S3.

**TABLE 1:** Diagnostic accuracy relative to the application of the modified BTPNA approach (tunnel lesions) with corresponding endosonographic signal extent, and non‐tunnel lesions influenced by the presence of a bronchus sign.

Type	Diagnostic accuracy	Number	*p*
Non‐tunnel lesions			
Overall	57.0%	130/228	0.35^a^
Bronchus sign	72.9%	78/107	0.59^b^
No bronchus sign	43.3%	52/121	0.06^b^
Tunnel lesions			
Overall	48.6%	17/35	0.35^a^
No/adjacent signal	29.4%	5/17	0.04^b^
SCP	66.7%	12/18	—

*Note*: Two tunnel lesions and seven non‐tunnel lesions were excluded for diagnostic accuracy estimation due to insufficient follow‐up data.

Abbreviation: SCP, semicircular to circular pattern.

^a^ Comparison of overall diagnostic accuracy of non‐tunnel lesions and tunnel lesions.

^b^ Compared to modified BTPNA approach with SCP signal.

### 3.3 Feasibility and Safety

A total of 37 attempts for tunnel creation were performed, of which 36 (97.3%) were successful. One attempt (2.6%) failed due to obstructive airways using a thin endoscope, occurring in a case targeting the right lower lobe. In two cases, insertion of R‐EBUS was unsuccessful due to the flexibility of the probe, resulting in an adjacent signal in one case and no signal in the other.

An UTB was utilised for tunnel creation in seven subjects, predominantly targeting lesions in the left upper lobe (57.1%). Endoscopic tunnel intubation was performed in 19 cases (51.4%), primarily with tunnel creation using a thin bronchoscope in 18 cases (94.7%) and an UTB in one case (14.3%, *p* < 0.05). Tunnel intubation was more commonly performed for lesions in the outer thoracic third compared to the non‐intubated group (47.4% vs. 11.8%, *p* = 0.04), which is also reflected in the shorter average distance to the pleura (26 ± 21.5 mm vs. 41.7 ± 18.4 mm, *p* = 0.03). It was also preferred for lesions located closer to the POE (12.3 ± 9 mm vs. 27.5 ± 16.5 mm, *p* < 0.01) Comprehensive details regarding bronchoscope size, examination data and endoscopic intubation are available in Tables S4–S6.

Pneumothorax occurred in 4 of 37 cases (10.8%), with a median chest tube duration of 6 (5.25–6.75) days. In 50% of these cases, cryobiopsy was used as a biopsy tool. These events were localised to the left upper lobe and the middle lobe. The distance from the lesion to the pleura was 15 mm, 16 mm, 26 mm, and 51 mm across the cases. Neither tunnel dilation using a balloon nor tunnel intubation with an UTB significantly impacted the pneumothorax rate (6.7% vs. 14.3% and 10.5% vs. 11.8%, respectively, for procedures with and without these interventions). There was one instance of moderate bleeding (≥ grade 2) with no occurrence of severe bleeding, intensive care requirements, or mortality. The median total bronchoscopy time was 78 (65.5–92.5) minutes including mediastinal staging, and the median post‐procedure hospital stay was 1 (1–3) day.

## 4 Discussion

This study presents a modified BTPNA approach (‘Essen Tunnel’) for lesions lacking direct airway access in virtual bronchoscopic navigation, where a vessel‐free tunnelling pathway can be established from a central, segmental, or subsegmental airway under endoscopic visualisation. This is the first description of the BTPNA approach via VBN without the use of a GS, optionally utilising an UTB for tunnel creation, followed by either R‐EBUS insertion or further endoscopic intubation.

The mBTPNA approach was successfully created in 97.3% of cases, demonstrating its technical feasibility. Notably, the use of an UTB enabled tunnelling through smaller and more distal airways, avoiding vascular structures and increasing the reach to lesions located in the outer third of the thoracic cavity. Furthermore, UTB intubation of the Essen Tunnel enabled sampling of more peripheral lesions with further distance from the POE. This demonstrates that the mBTPNA technique provides access to intraparenchymal lesions across diverse locations, overcoming the technical limitations of traditional BTPNA methods, which rely on the use of a guide sheath and are thus restricted by the limited selection of compatible bronchoscope sizes. The technique proved particularly advantageous for lesions in the left upper lobe, a region where traditional BTPNA approaches are often complicated by central vascular anatomy [17]. This ability to bypass vascular structures and access distal airways broadens the scope of targetable lesions. The mBTPNA technique is particularly beneficial for lesions in the central or middle thorax, where transthoracic biopsy carries a higher pneumothorax risk due to deep tissue penetration [1, 2]. It is also a valuable alternative when bone structures, such as the scapula, obstruct transthoracic access [17], provided the necessary expertise and technical infrastructure are available. In the first BTPNA trial, a tunnel length of 50 mm or longer was achieved in seven out of ten patients, with a 90‐mm long tunnel successfully created in one patient [13]. Sun et al. reported no significant difference in biopsy yield between tunnel lengths of < 30 mm and ≥ 30 mm [14]. In our study, the tunnel length did not exceed 35 mm. Consequently, we are unable to draw definitive conclusions regarding the feasibility and safety of the mBTPNA approach for longer tunnel lengths. Further evaluation and validation of this approach are necessary in applicable cases.

R‐EBUS serves as a valuable adjunct by providing real‐time imaging information for diagnosing lesions [26]. A key finding from our study is that integrating R‐EBUS into BTPNA significantly improved the endosonographic lesion detection rate and, importantly, despite the challenging nature of intraparenchymal pulmonary lesions, SCP or circular signals reached high diagnostic yields, making it comparable to that of lesions exhibiting a bronchus sign. In some cases, despite the initial presence of at least a semicircular endosonographic signal, technical challenges in accurately targeting the lesion at the airway level necessitated tunnel creation from more centrally accessible endoscopic positions to optimise biopsy conditions and achieve a broader extension of the lesion signal. One advantage of BTPNA procedures is the ability to perform cryobiopsies on pulmonary nodules without a direct airway connection. While cryobiopsy showed higher diagnostic accuracy, the difference was not statistically significant, likely due to limited statistical power. Its diagnostic value for peripheral nodules is well established [27, 28, 29], but its role in BTPNA requires further validation in future studies. In comparison to data from Sun et al., who reported an 86.3% biopsy yield for Guided TBNA and BTPNA, the majority of their lesions (81.2%) exhibited a bronchus sign or had a direct airway leading to the lesion, and 60.5% were larger than 2 cm. In contrast, our cohort represents a significantly more challenging group, as it primarily consisted of lesions smaller than 2 cm, with a notably lower proportion showing a bronchus sign [14]. Emerging technologies such as cone beam CT [30, 31] are promising for further enhancing the precision of BTPNA approaches by addressing CT‐to‐body divergence issues like anaesthesia‐induced atelectasis and verification of tools in lesion confirmation during biopsy [32, 33].

The omission of a GS in the mBTPNA technique offers several advantages. The currently available GS is incompatible with ultrathin bronchoscopes, restricting tunnelling in peripheral regions. Additionally, the GS is relatively rigid and cannot be adjusted once it exits the bronchoscope's working channel. In contrast, advancing an ultrathin bronchoscope through the tunnel allows for greater flexibility by enabling angulation and fine‐tuned adjustments of the bronchoscope tip. From a procedural perspective, omitting the GS simplifies workflow and optimises resource efficiency. Furthermore, the application of R‐EBUS is feasible within this technique. However, GS placement may provide tamponade within the intraparenchymal tunnel, potentially mitigating bleeding risks. Nevertheless, ensuring a vessel‐free tunnelling pathway using VBN‐Doppler and R‐EBUS should help minimise the likelihood of vascular injury. In our study cohort, only one case of Grade II bleeding was observed, which was successfully controlled with suctioning and topical vasoconstrictor application. Therefore, our findings do not indicate an increased bleeding risk associated with a GS‐free workflow. Further studies are required to systematically assess the benefits and potential limitations of GS usage in mBTPNA and its impact on procedural safety.

The pneumothorax rate for the Essen Tunnel was 10.8%, aligning with previous studies of traditional BTPNA techniques, most of which are pilot studies with small sample sizes: in the first in‐human study by Herth et al., tunnel creation succeeded in 83.3% (10/12) of cases, but the subsequent lobe resection precluded a reliable assessment of the safety profile of the procedure [13]. A later study concluded that BTPNA is feasible in the setting of an endoscopy unit, with successful tunnelling in 5 of 6 lesions. Subjects were followed for adverse events for 72 h, with pneumothorax occurring in 40% (2 out of 5) and one case requiring a chest tube [34]. Herth et al. compared different hospital types regarding BTPNA safety and feasibility. Adverse events occurred in 11.1% of cases in training hospitals and 7.6% in community hospitals [35]. In our study, Cryobiopsy was performed in 50% of pneumothorax cases, which likely contributed to the observed rate, given its known pneumothorax risk of approximately 20% [36, 37]. The increased pneumothorax incidence with mBTPNA may also be influenced by the use of UTB for tunnel creation and the frequent proximity of lesions to the pleura in these cases. Hazheim et al. reported pneumothorax in two out of six patients, both with a short lesion‐to‐pleura distance (1 mm and 9 mm, respectively), reinforcing the importance of this factor [34]. Zhang et al. similarly highlighted this risk [17], underscoring the need for further clinical investigation. Patients undergoing mBTPNA with peripheral lesion locations and proximity to the pleura, particularly when cryobiopsy is performed, should be carefully clinically evaluated for the risk of pneumothorax. Future studies with larger datasets are essential to validate this association. Overall, the risk of pneumothorax after preparation of an Essen Tunnel was still favourable over transthoracic approaches [1, 2, 38], where the risk increases with the depth of transthoracic needle insertion, particularly centrally or in intermediately located nodules [1, 2], which were predominantly present in our cohort. Furthermore, the risk of bleeding was low in our study cohort, most likely due to the use of VBN‐Doppler and R‐EBUS to visualise vascular structures along the tunnel path. Importantly, mBTPNA did not prolong hospital stay, with a median discharge time of 1 day after the intervention, which aligns well with the trend towards shorter hospital stays and increased ambulatory care [39, 40]. Although this was not included in our statistical analysis, a reduction in material consumption was observed when the BTPNA‐Access Kit, including the GS, was not used for tunnel creation, which is consequently associated with lower procedural costs.

However, this study has several limitations. Being a single‐centre study conducted in a highly specialised lung centre, the generalizability of the findings to less experienced settings may be limited. The retrospective design introduces potential biases, such as selection bias. The relatively small sample size of lesions targeted with the Essen Tunnel compared to non‐tunnel lesions reduces statistical power. The influence of operator experience was not assessed, leaving uncertainty regarding the learning curve required for successful implementation. Furthermore, the use of cryobiopsy, known to carry a higher pneumothorax risk, may have inflated the complication rate, and the choice of biopsy technique was not standardised. Furthermore, the reported bronchoscopy duration includes the entire procedure rather than specifically isolating the time required for the mBTPNA technique. Certain complications, such as infections, may be underrepresented in the dataset because they may have occurred delayed and were therefore not recorded during the hospitalisation period. Future prospective studies are needed to address these limitations and confirm the technique's utility across diverse clinical settings.

In conclusion, the modified BTPNA technique, known as the ‘Essen Tunnel’, has proven to be a feasible approach that significantly enhances endosonographic lesion detection and improves diagnostic accuracy for challenging intraparenchymal lesions. When endosonography detects a semicircular or circular pattern through the tunnel, the results are comparable to those seen in lesions with a bronchus sign. The use of an ultrathin bronchoscope for tunnel creation and subsequent intubation further expands lesion accessibility. The technique demonstrates a favourable safety profile.

## Author Contributions

**Erik Büscher:** conceptualization (equal), data curation (equal), formal analysis (equal), methodology (equal), visualization (equal), writing – original draft (lead), writing – review and editing (equal). **Faustina Funke:** conceptualization (equal), investigation (equal), methodology (equal), supervision (equal), writing – original draft (equal), writing – review and editing (equal). **Jane Winantea:** data curation (equal), investigation (equal), writing – review and editing (equal). **Hanna Zellerhoff:** data curation (equal), investigation (equal), writing – review and editing (equal). **Johannes Wienker:** data curation (equal), writing – review and editing (equal). **Marcel Opitz:** data curation (equal), writing – review and editing (equal). **Christian Taube:** data curation (equal), writing – review and editing (equal). **Kaid Darwiche:** conceptualization (equal), investigation (equal), methodology (equal), resources (lead), supervision (equal), writing – original draft (equal), writing – review and editing (equal).

## Ethics Statement

The study received approval from the Ethics Committee of the Medical Faculty of the University of Duisburg Essen (24‐11881‐BO).

## Conflicts of Interest

Faustina Funke received speaker honoraria from Broncus Medical. Kaid Darwiche received travel support and research grants from Broncus Medical. The other authors declare no conflicts of interest.

## Supporting information


**Data S1.** Supporting Information.

## Data Availability

The data that support the findings of this study are available from the corresponding author upon reasonable request.
