# Supplementary material for: Feasibility of a Modified Bronchoscopic Transparenchymal Nodule Access Technique (‘Essen Tunnel’) for Improving the Diagnosis of Intraparenchymal Pulmonary Lesions
Source: Respirology. 2025 May 21;30(9):851–60. doi: 10.1111/resp.70053 (PMC12438015; doi:10.1111/resp.70053)
Supplement: Supplementary file 1 — Data S1. Supporting Information. [file RESP-30-851-s001.docx]

**Feasibility of a Modified Bronchoscopic Transparenchymal Nodule Access Technique (“Essen Tunnel”) for Improving the Diagnosis of Intraparenchymal Pulmonary Lesions**

Authors: E. Büscher^1*^, F. Funke^1*^, J. Winantea^1^, H. Zellerhoff^1^, J. Wienker^1^, M. Opitz^2^, C. Taube^3^, K. Darwiche^1^

*Shared first authorship

**Supplement**

Affiliations:

^1^ Department of Pulmonary Medicine, Section for Interventional Pneumology, Ruhrlandklinik University Hospital Essen - Essen (Germany),

^2^ Institute of Diagnostic and Interventional Radiology and Neuroradiology, University Hospital Essen - Essen, Germany.

^3^ Department of Pulmonary Medicine, Ruhrlandklinik, University Hospital Essen - Essen (Germany)

**Table S1** Comparison of baseline characteristics and lesion parameters with the Essen Tunnel Approach***** Abbreviations: BMI = Body Mass Index, ECOG = Eastern Cooperative Oncology Group performance status, IVC = Inspiratory Vital Capacity, FEV1 = Forced Expiratory Volume in 1 second, DLCO = Diffusing Capacity of the Lung for Carbon Monoxide, COPD = Chronic Obstructive Pulmonary Disease, RUL = Right upper lobe, RML = Right middle lobe, RLL = Right lower lobe, LUL = Left upper lobe, LLL = Left lower lobe, les. = lesions; missing values:^1^ in 2 subjects with tunnel lesion, ^2^ in 2 tunnel lesions and 6 non-tunnel lesions ^3^ in 4 non-tunnel lesions ^4^ in 1 tunnel lesion and 11 non-tunnel lesions;

|  | **Tunnel les.**, n = 37 | **Non-tunnel les**., n = 230 | **P-Value** |
| --- | --- | --- | --- |
| **Baseline characteristics** | | | |
| Subjects, n | 37 | 220 |  |
| Gender, female | 21 (56.8 %) | 113 (51.4 %) | 0.37 |
| Age, years | 68.4 ± 10.6 | 67.1 (± 10.3) | 0.49 |
| BMI | 25.8 ± 4.7 | 25.7 ± 5.5 | 0.88 |
| Smoker/former Smoker, n | 30 (81.1 %) | 184 (83.6 %) | 0.70 |
| **Pulmonary function, predicted %** | |  |  |
| IVC^1^ | 84.3 ± 20.8 | 86.6 ± 19.7 | 0.76 |
| FEV1^1^ | 68 ± 21.9 | 67.2 ± 25.8 | 0.83 |
| DLCO^2^ | 57.5 ± 22.2 | 54.1 ± 24.0 | 0.45 |
| **Lesions parameter** | | | |
| Bronchus sign | 12 (32.4 %) | 108 (47 %) | 0.10 |
| Diameter, long axis in mm^3^ | 18.6 ± 6.5 | 20.8 ± 8.3 | 0.12 |
| Diameter long axis < 2cm^3^ | 22 (59.5 %) | 131 (57 %) | 0.86 |
| Target length, mm^4^ | 12.7 ± 4.1 | 13.8 ± 5.9 | 0.39 |
| Distance to target, mm^4^ | 20.3 ± 15.3 | 17.2 ± 13.2 | 0.28 |
| Dist. from les. - pleura, mm^4^ | 34.4 ± 22.3 | 18.8 ± 18.2 | <0.01 |
| Lobe distribution, n (%) |  |  | 0.84 |
| RUL | 11 (29.7 %) | 74 (32.2 %) |  |
| RML | 3 (8.1 %) | 11 (4.8 %) |  |
| RLL | 9 (24.3 %) | 46 (20.0 %) |  |
| LUL | 9 (24.3 %) | 70 (30.4 %) |  |
| LLL | 5 (13.5 %) | 29 (12.6 %) |  |
| Thoracic site of the lesion   - peripheral - intermediate - central | 12 (32.4 %)  23 (62.2 %)  2 (5.4 %) | 139 (60.4 %)  85 (37 %)  6 (2.6 %) | 0.01 |

**Table S2**: Endosonographic lesion signal categories before and after tunneling, with data presented for all subjects in the left half of the table and exclusively for subjects with R-EBUS insertion in the right half.

| **Endosographic signal category** | **All subjects (n = 37)** | | | **Subjects with R-EBUS Insertion (n = 31)** | | |
| --- | --- | --- | --- | --- | --- | --- |
|  | Pre-Tunneling,  n = 37 | Post-Tunneling,  n = 37 | P-Value | Pre-Tunneling | Post-Tunneling, |  |
| No signal | 67.6 % (25/37) | 37.8 % (14/37) | <0.01 | 61.3 % (19/31) | 25.8 % (8/31) | <0.01 |
| Adjacent Signal | 16.2 % (6/37) | 10.8 % (4/37) | 0.625 | 19.4 % (6/31) | 12.9 % (4/31) | 0.625 |
| Semicircular Signal | 13.5 % (5/37) | 24.3 % (9/37) | 0.344 | 16.1 % (5/31) | 29.0 % (9/31) | 0.34 |
| Three-quarter Signal | 0.0 % | 8.1 % (3/37) | - | 0.0 % | 9.7 % (3 /31) | - |
| Circular Pattern | 2.7 % (1/37) | 18.9 % (7/37) | 0.13 | 3.2 % (1/31) | 22.6 % (7/31) | 0.13 |
| Semicircular to circular pattern | 16.2 % (6 / 37) | 51.4 % (19/37) | <0.01 | 19.4 % (6/31) | 61.3 % (19/31) | <0.01 |

**Table S3** Diagnostic accuracy, including cases lost to follow-up, was assessed relative to the application of the modified BTPNA approach (tunnel lesions) in correlation with the extent of endosonographic signal and non-tunnel lesions influenced by the presence of a bronchus sign. * Comparison of overall diagnostic accuracy of non tunnel lesions and tunnel lesions; ** Comparison with the modified BTPNA approach showing an SCP signal; SCP = semicircular to circular pattern.

| **Type** | Diagnostic accuracy | Number | P-Value |
| --- | --- | --- | --- |
| **Non-Tunnel Lesions** | | | |
| Overall | 55.3 % | 130 / 235 | 0.29* |
| Bronchus sign | 70.9 % | 78 / 110 | 0.49** |
| No Bronchus sign | 41.6 % | 52 / 125 | 0.08** |
| **Tunnel Lesions** | | | |
| Overall | 45.9 % | 17 / 37 | 0.29* |
| No Signal | 27.8 % | 5 / 18 | 0.04** |
| SCP | 63.2 % | 12 / 19 | - |

**Table S4** Lesion characteristics and feasibility based on bronchoscope size, R-EBUS = radial endobronchial ultrasound; No. = number; RUL = right upper lobe; RML = right middle lobe; RLL = right lower lobe; LUL = left upper lobe; LLL = left lower lobe; interm. = intermediate, Ptx = Pneumothorax, n.s. = not significant; ^1^One instance of missing data for a lesion targeted using a ultrathin bronchoscope

|  | **Bronchoscope size** | |  |
| --- | --- | --- | --- |
|  | Thin (4.2 mm), n = 30 | Ultrathin (3mm), n = 7 | P-Value |
| Technical feasible | 29 (96.6 %) | 7 (100 %) | n.s. |
| R-EBUS inserted | 24 (80 %) | 7 (100 %) | n.s. |
| Endoscope intubation | 18 (60 %) | 1 (14.3 %) | 0.04 |
| Cryobiopsy used | 8 (26.7 %) | 4 (57.1 %) | n.s. |
| Target length, mm^1^ | 12.4 ± 3.5 | 12.7 ± 4.1 | n.s. |
| Distance to target, mm^1^ | 21.1 ± 16.1 | 16.3 ± 11.2 | n.s. |
| Distance to Pleura, mm^1^ | 35.2 ± 24 | 30.3 ± 11.7 | n.s. |
| Occurrence of Ptx | 1 (3.3 %) | 3 (42.9 %) | 0.02 |
| Lobe distribution |  |  | 0.01 |
| RUL | 11 (36.7 %) | 0 |  |
| RML | 1 (3.3 %) | 2 (28.6 %) |  |
| RLL | 9 (30.0 %) | 0 |  |
| LUL | 5 (16.7 %) | 4 (57.1 %) |  |
| LLL | 4 (13.3 %) | 1 (14.3 %) |  |
| Thoracic site of the lesion |  |  | n.s. |
| peripheral | 10 (33.3 %) | 2 (28.6 %) |  |
| interm./central | 20 (66.7 %) | 5 (71.4 %) |  |

**Table S5** Investigation-specific data; diag. = diagnostic, min = minutes, therap. = therapeutic, w. = with, *Grade 2 on Nashville working group, ^1^ One lesion with missing data.

| **Investigation** |  |
| --- | --- |
| Bronchoscope, thin. / ultrathin., n | 30 (81,1 %) / 7 (19.9 %) |
| Virtual target reached, n | 27 (73.0 %) |
| Ballon dilatation, n | 17 (45.9 %) |
| R-EBUS introduced | 31 (83.8 %) |
| - No signal - Adjacent signal - Semicircular (90° - 240°) - Circular° | - 8 (25.8 %) - 4 (12.9 %) - 14 (45.2 %) - 5 (16.1 %) |
| Tunnel Intubation w. endoscope, n | 19 (51.4 %) |
| Sampling with |  |
| - Forceps, n - Cryobiopsy - Needle - Catheter - Brush | - 30 (81.1 %) - 12 (32.4 %) - 28 (75.7 %) - 7 (18.9 %) - 3 (8.1 %) |
| Planned tunnel length^1^ | 20.3 ± 15.3 |
| Median Bronchoscopy duration, min | 78 (65.5 - 92.5) |
| Median Stay in Recovery room, min | 68 (49.5 - 78.5) |

**Table S6:** Lesion characteristics and feasibility based on endoscope intubation, R-EBUS = radial endobronchial ultrasound; No. = number; RUL = right upper lobe; RML = right middle lobe; RLL = right lower lobe; LUL = left upper lobe; LLL = left lower lobe; interm. = intermediate. ^1^One lesion without endoscope intubation with missing data

|  | **Endoscope intubation** | |  |
| --- | --- | --- | --- |
|  | Not performed, n = 17 | Performed, n = 19 | Significance |
| Target length*, mm^1^ | 13.1 ± 5.1 | 12.2 ± 3.2 | n.s. |
| No. of branches^1^ | 5 (5 - 6.75) | 6 (5 - 8) | n.s. |
| Distance to target, mm^1^ | 12.3 ± 9.0 | 27.5 ± 16.5 | < 0.01 |
| Distance to Pleura, mm^1^ | 41.7 ± 18.4 | 26 ± 21.5 | 0.03 |
| Lobe distribution, n |  |  | n.s. |
| RUL | 4 (23.5 %) | 7 (36.8 %) |  |
| RML | 3 (17.6 %) | 0 |  |
| RLL | 4 (23.5 %) | 4 (21.1 %) |  |
| LUL | 4 (23.5 %) | 5 (26.3 %) |  |
| LLL | 1 (11.8 %) | 3 (15.8 %) |  |
| Thoracic site of the lesion |  |  | 0.04 |
| peripheral | 2 (11.8 %) | 9 (47.4 %) |  |
| interm./central | 14 (82.4 %) | 10 (52.6 %) |  |
